# Supplementary material for: Aetiology, antimicrobial susceptibility patterns and factors associated with bacteriuria among HIV-infected women attending Prevention of Mother-to-Child Transmission (PMTCT) clinic at Bukoba Municipality, Tanzania
Source: PLoS One. 2025 Mar 19;20(3):e0311474. doi: 10.1371/journal.pone.0311474 (PMC11922243; doi:10.1371/journal.pone.0311474)
Supplement: Attachment — Submitted filename: Response to Reviewers.docx [file pone.0311474.s003.docx]

**RESPONSE TO REVIEWER’S COMMENTS**

**Title:** Aetiology, antimicrobial Susceptibility Patterns and factors associated with bacteriuria among HIV-infected women attending Prevention of Mother to Child Transmission Clinic at Bukoba Municipality, Tanzania

**Editor Comments**

Thank you for stating the following financial disclosure: We received a small fund for data collection from TFELTP. Please state what role the funders took in the study. If the funders had no role, please state: "The funders had no role in study design, data collection and analysis, decision to publish, or preparation of the manuscript."

**Response:** The statement has been added to the cover letter to show that the funders had no role in study design, data collection and analysis, decision to publish, or manuscript preparation.

We note that your Data Availability Statement is as follows: All relevant data are within the manuscript and its Supporting Information files. Please confirm at this time whether or not your submission contains all raw data required to replicate the results of your study. Authors must share the “minimal data set” for their submission. PLOS defines the minimal data set to consist of the data required to replicate all study findings reported in the article, as well as related metadata and methods (<https://journals.plos.org/plosone/s/data-availability#loc-minimal-data-set-definition>).

**Response:** - We confirm that the submission contains all raw data required to replicate the result of the study

When completing the data availability statement of the submission form, you indicated that you will make your data available on acceptance. We strongly recommend all authors decide on a data sharing plan before acceptance, as the process can be lengthy and hold up publication timelines. Please note that, though access restrictions are acceptable now, your entire data will need to be made freely accessible if your manuscript is accepted for publication. This policy applies to all data except where public deposition would breach compliance with the protocol approved by your research ethics board. If you are unable to adhere to our open data policy, please kindly revise your statement to explain your reasoning and we will seek the editor's input on an exemption. Please be assured that, once you have provided your new statement, the assessment of your exemption will not hold up the peer review process.

**Response:** The data is available and agree to provide the necessary information whenever needed, but if the exemption is accepted I will appreciate it.

PLOS requires an ORCID iD for the corresponding author in Editorial Manager on papers submitted after December 6th, 2016. Please ensure that you have an ORCID iD and that it is validated in Editorial Manager. To do this, go to ‘Update my Information’ (in the upper left-hand corner of the main menu), and click on the Fetch/Validate link next to the ORCID field. This will take you to the ORCID site and allow you to create a new iD or authenticate a pre-existing iD in Editorial Manager.

**Response:** The ORCID ID has been updated on my account. The ORCID ID is 0000-0002-5793-2867

One of the noted authors is a group or consortium. In addition to naming the author group, please list the individual authors and affiliations within this group in the acknowledgments section of your manuscript. Please also indicate clearly a lead author for this group along with a contact email address.

**Response:** The acknowledgment section and the leading author have been indicated together with a contact email address.

Please amend either the abstract on the online submission form (via Edit Submission) or the abstract in the manuscript so that they are identical.

**Response:** The abstract on the online submission form has been amended to align with the abstract in the manuscript so that they are identical

Please review your reference list to ensure that it is complete and correct. If you have cited papers that have been retracted, please include the rationale for doing so in the manuscript text, or remove these references and replace them with relevant current references. Any changes to the reference list should be mentioned in the rebuttal letter that accompanies your revised manuscript. If you need to cite a retracted article, indicate the article’s retracted status in the References list and also include a citation and full reference for the retraction notice.

**Response:** The reference has been revised.

**Additional Editor Comments**

When submitting the revised manuscript, please ensure that line numbers and page numbers are included for ease of reference in the review process

**Response:** Line numbers and page numbers have been added.

In the Introduction section, please define PMTCT upon its first mention and clarify any other abbreviations that have not been previously defined

**Response:** We have defined PMTCT and other abbreviations upon their first mention Page 4, L110

In the discussion section, please provide an interpretation of your study results, discussing the implications of these findings in the context of similar studies in the literature. Avoid repeating results in this section.

**Response:** The discussion section has been revised to accommodate the suggestion. Page ---, lines -----

Remove the sub-headings "Limitations" and "Recommendations." The limitations of the study should be detailed in the final paragraph of the Discussion section, while recommendations based on your findings should appear as the last paragraph of the Conclusion section.

**Response:** Subheadings for limitations and recommendations have been removed and the last paragraph of the discussion and conclusion revised accordingly. Page 17 L448-468.

Ensure that bacterial names are consistently italicized throughout the manuscript. Specifically, please italicize E. coli in the Conclusion section.

**Response:** We have reviewed the entire manuscript and consistently italicized the bacterial names including *E. coli* in the conclusion section. Pg 17, L455

**REVIEWER #1**

The study offers valuable insights into the high prevalence of bacteriuria and alarming levels of antibiotic resistance among HIV-infected women in a resource-limited setting. The findings align with regional studies; however, the absence of significant associations with bacteriuria risk factors indicates a need for more comprehensive investigations to identify potential predictors and effectively target interventions. The identification of a high proportion of multidrug-resistant (MDR) pathogens (68.2%) raises concern. It underscores the urgent need for improved antimicrobial stewardship, rational antibiotic use, and enhanced infection control measures to combat antibiotic resistance in vulnerable populations.

**Response:** Thank you for your comment; we have taken some comments as recommendations for further study.

The data and analyses robustly support the claims, and the described methodology is sufficient for reproducing the experiments. The authors appropriately contextualize their claims within the existing literature, fairly treating previous studies. They compare the prevalence of bacteriuria and MDR pathogens in their research with findings from similar studies conducted in Iran, Ethiopia, Nepal, Nigeria, and other regions of Tanzania, acknowledging geographical, methodological, and healthcare delivery differences that may explain variations in results

**Response:** Thank you for the comments

While the study effectively highlights high resistance rates linked to irrational use and easy access, incorporating clinical data on antibiotic use within and outside the PMTCT clinic could further enhance the analysis. This addition would facilitate correlations between resistance patterns and factors such as age, socioeconomic status, or prior antimicrobial use, leading to a more nuanced understanding of AMR drivers and improving the study's capacity to inform targeted interventions.

**Response:** This is well noted and appreciated, but during data collection, we didn’t collect data outside the PMTCT clinic, so it was hard to conduct analysis and make comparisons.

The authors' recommendations serve as a good starting point but could be strengthened for greater impact. Emphasizing the importance of continuous education for healthcare providers on antibiotic stewardship would enhance their recommendations. Additionally, defining rational use in the context of local resistance patterns and incorporating strategies for infection prevention and control would create a more comprehensive approach to addressing antimicrobial resistance.

**Response:** We have revised the recommendations emphasizing a need for education to healthcare providers on antibiotic stewardship, IPC, and rational use of antibiotics. Page 19, L463-466

The write-up could also benefit from minor edits to correct organization and typographical errors for improved clarity.

**Response:** The entire manuscript has been reviewed for typographical/grammatical errors.

**REVIEWER #2**

In the title, instead of “prevention of mother to child transmission” capitalize the first words of the clinic name “Prevention of Mother to Child Transmission (PMTCT)”.

**Response:** Thank you for the comment, the first words have been capitalized as suggested. page 1, L1-3

While the ethical considerations are well addressed, including information about how confidentiality was maintained during the study would strengthen this section.

**Response:** The information about the confidentiality of participants has been added, to page 8 L246-250

**REVIEWER #3**

The in-text citations are inconsistent (a mix of round and square brackets). Please note that PLOS One requires the use of reference numbers in square brackets, so this needs to be corrected throughout the manuscript.

**Response:** The square brackets have been used throughout the manuscript in reference citation.

Include page numbers and line numbers in the submitted manuscript for easier reference.

**Response:** Page and line numbers have been included in the manuscript

Ensure that the spacing between paragraphs is consistent across the document.

**Response:** We have checked the whole document to ensure that the spacing between paragraphs is consistent.

Under the “Financial Disclosure” section, please write out the full name of the funding body, specifically for TFELTP.

**Response:** The full name of the funding body TFELTP has been written in the financial disclosure section

The corresponding author list should not include physical addresses; only email addresses are required. List the corresponding author’s initials in parentheses after the email address. (See PLOS One Title, Author, Affiliations Formatting Guidelines)

**Response:** The physical address of the corresponding author has been removed, and the list of authors has been written according to PLOS ONE guidelines. Page 1

**Abstract**

1. In the sentence “17 (34.0%) of gram-negative bacteria were Extended Spectrum Beta Lactamase (ESBL) procedures,” please clarify if "procedures" is the intended term, as it seems out of context. Consider revising for clarity.

**Response:** The intended word was “producers”. The statement has been revised for clarity. Page1, L41.

2. In the phrase “Escherichia coli showed high rate of resistance rate against trimethoprim-sulfamethoxazole 21(100%),” please choose one term for consistency. For example, use "high rate of resistance" or "resistance rate" but not both.

Response: The phrase has been revised by choosing “high rate of resistance” for consistency. Page 2, L42

3. The statement under Recommendations is vague. The authors should specify whether they are referring to prescribed or over-the-counter antimicrobial agents.

**Response:** The recommendations have been revised for clarity. Page 2, L50-51

**Introduction**

Paragraph 3: Please state the country in which Mwanza is located for clarity.

**Response:** The country (Tanzania) where Mwanza is located has been added. Page 3, L73

Paragraph 4: Ensure that genus/species names are written in italics to adhere to scientific formatting conventions.

**Response:** Genus/species name has been written in italics to adhere to scientific formatting. Page 3

**Material And Methods**

Urine specimen collection and handling: 10 ml not ten mL

**Response:** Corrections has been made. Page 5, L145

Reference 28 is incorrectly cited. This study examined the effect of handwashing with water or soap and did not involve the use of a sterile cotton swab soaked in normal saline to reduce contamination. Therefore, this reference should be placed after "soap" for accurate attribution.

**Response:** We have corrected the position of reference 28, as suggested. Page 5, L148

Urine culture and bacteria identification: It would be great if the authors could state why a hydrogen sulfide production test was performed when the Kligler Iron Agar tests for hydrogen sulfide production.

**Response:** According to the laboratory SoP, both SIM and KIA tests are used to identify gram-negative bacteria; hydrogen sulfide is tested on methods. Page 6, L162-165

**Results**

Socio-demographic and clinical characteristics of the study participants: There is a lack of agreement between the data in Table 2 and the accompanying text (See the last line under this section of the manuscript).

**Response:** We have noted the lack of agreement between the data in the table and text. We have corrected the table. Page 8-9, L255-267

Table 3: Isolates “genus and species name” should be in italics.

**Response:** The genus and species name in Table 3 have been written in italics. Page 11,

The letter case for the acronym for Coagulase Negative Staphylococcus should be consistent throughout the text.

**Response:** The acronym for Coagulase Negative Staphylococcus has been revised to be consistent throughout the text.

Antimicrobial resistance of isolated bacteria from HIV-infected women attending PMTCT clinic at Bukoba Municipality: Check Line 4 “….and 3(14.0%) for nitrofurantoin (Table 4)”. The correct table reference for nitrofurantoin is Table 3, not 4.

Response: Thanks for the comment. This has been updated from table 4 to table 3. Page 11

Factors associated with bacteriuria in HIV-infected women attending PMTCT clinic at Bukoba Municipality: Check the numbering of the tables-Table 6, not Table 2

**Response:** The table number has been revised from table 2 to table 6.

Formatting of Table 6 (wrongly titled as Table 2): The table is incorrectly titled Table 2. Please correct the title to Table 6. The table exhibits inconsistent line and column formatting. Ensure that the text within the table follows a uniform layout for clarity and readability. The table suffers from inconsistent line/column formatting. The text in the table should follow a uniform layout.

**Response:** We corrected the table number to Table 6 and formatted the lines and columns to exhibit consistency. Pages 16 and 17.

**Discussion**

The authors should elaborate more on the multivariable modified Poisson regression analysis for bacteriuria (see last paragraph in the discussion section)

Response: We have added the description to elaborate more on the multivariable modified Poisson regression analysis for bacteriuria. Page 16, L439-445

**Conclusions:**

The authors should include potential future directions for research.

**Response:** Potential future directions for research have been added. Page 17, L458

Line 1: “E. coli” should be in italics

**Response*:*** *E. coli* in conclusion has been written in italics form. Page 17, L455

**Study Limitations**

Participants may experience recall bias. If they were unable to remember all relevant information during data collection, this limitation should be acknowledged in the discussion section.

**Response:** The limitation of recall bias has been acknowledged in the discussion section. Page 16, L150

**Recommendations**

I recommend merging the recommendations with the conclusions section to create a more cohesive final study summary. Also, correct the “National Essential Medicines List (STG/NEMLIT) to avoid rational antimicrobial therapies.” Check the usage of “rational” in this context.

**Response:** Recommendations have been merged with conclusions, and the word rational has been revised. Page 17, L461-468

**Availability of data and material**

PLOS journals require authors to make all data necessary to replicate their study’s findings publicly available without restriction at the time of publication. Stating ‘data available upon request from the corresponding author’ is insufficient.

**Response:** We have shared the required data as a supplementary file.

**Supplementary materials**

The authors should include pictures of the media plates used in the study. Additionally, data measuring the zones of inhibition for the antimicrobial susceptibility testing (AST) should be provided.

**Response:** Unfortunately, we did not take pictures of the media plates used in the study. For the zones of inhibition for AST, we used breakpoint as per CLSI guidelines, reference number 31.

**Reference**

The title of the research article in Reference 2 should be included for completeness.

**Response:** The title of the research article in reference 2 has been added. Page 18, L497-499

For Reference 7, please ensure the author's name is formatted in sentence case to comply with the citation guidelines.

**Response:** The author’s name in reference 7 has been formatted in sentence case. Page 19, 515

**REVIEWER #4:**

The title should be “Aetiology, antimicrobial susceptibility patterns and factors associated with bacteriuria among HIV-infected women attending the Prevention of Mother-to-Child Transmission (PMTCT) clinic at Bukoba Municipality, Tanzania.”

**Response:** We have revised the title as suggested, Page 1, lines 1-4

Did you want to study asymptomatic bacteriuria, as defined in your introduction, or symptomatic bacteriuria, which is UTI? You cannot define bacteriuria as “without the presence of symptoms” if there are two types.

**Response:** The study involved both symptomatic and asymptomatic bacteriuria. We have rephrased the statement for clarity. Page 1, Line 23

Pregnant women living with HIV belong to a defined population. Why did you choose them? What is your rationale? Are there any risks associated with bacteriuria (symptomatic or not) in them? Why is this of concern to them and public health in general?

**Response:** This is elaborated on in the introduction. The human immunodeficiency virus (HIV) in both pregnant and lactating women has a high chance of acquiring opportunistic infections, including UTIs, especially in developing countries with limited health services. Page 2 to 4

The title of your study does not include lactating women living with HIV. They are brought into the write-up somewhere in the middle and not mentioned in the discussion and conclusion. Were they important to this study in the first place? Why is bacteriuria (symptomatic or not) important in these women? What is the importance of lactation to bacteriuria?

**Response:** At PMTCT, they attend both pregnant and lactating women living with HIV. Lactating women were not studied in the first place; we thought to include them. The study is important as we presented the findings with significance corresponding with the study area.

Informed consent is built on the voluntary agreement of people to participate in a study after every relevant piece of information has been made available to them. Convincing them erodes their freedom to participate and deviates from the ethics of research involving humans. Did you explain the study in detail and allow them to choose to participate or did you convince them as you have written?

**Response:** The study was explained to participants in detail, including the benefits and risks, and allowed them to choose whether to participate or not. We have rephrased the statement in the sampling technique subsection for clarity.

Your discussion should be in shorter paragraphs, each addressing a specific finding. This will make it easier to follow your points.

**Response:** We have revised the discussion section for each paragraph to address specific findings

Your manuscript is titled “… and factors associated with bacteriuria among HIV-infected women …” but at the end of your discussion, you declared that no factors were associated with bacteriuria in this study. If you aimed to demonstrate the significance or otherwise of pre-defined factors to the incidence of bacteriuria in this population, then it would be acceptable to declare that you found no association between the variables but this is not the case here. In your results, you will find some factors associated with a higher incidence of bacteriuria in the population you studied. Discuss them. They do not necessarily have to be causative.

**Response:** We discussed some factors associated with a higher incidence of bacteriuria.

I am sure that you want your study to be relevant to the care and management of pregnant women living with HIV. How does your study contribute to the prevention and control of bacteriuria in these women? You should include this in your conclusion and recommendations.

**Response:** The study’s contribution to the prevention and control of bacteriuria has been included in the recommendation.

Your recommendations address the judicious use of antibiotics but is that all there is to prevent and control bacteriuria in this population if it is important enough to be studied?

**Response:** Through our study and based on study populations, settings, practices, and policies of our country, we thought this recommendation might help.
